# Single-stapling *versus* double-stapling technique for rectal anastomosis—meta-analysis

**DOI:** 10.1093/bjsopen/zrag078

**Published:** 2026-07-03

**Authors:** Dimitrios Kehagias, Charalampos Lampropoulos, Ioannis Kehagias, David Jayne, Jim Tiernan

**Affiliations:** John Goligher Colorectal Unit, Leeds Teaching Hospitals NHS Trust, Leeds, UK; Intensive Care Unit, St Andrew’s General Hospital of Patras, Patras, Greece; Department of Surgery, University Hospital of Patras, Patras, Greece; John Goligher Colorectal Unit, Leeds Teaching Hospitals NHS Trust, Leeds, UK; Leeds Institute of Medical Research, University of Leeds, Leeds, UK; John Goligher Colorectal Unit, Leeds Teaching Hospitals NHS Trust, Leeds, UK

**Keywords:** rectal cancer, anastomotic leak, transanal transection and single-stapled anastomosis (TTSS)

## Abstract

**Introduction:**

The single-stapling technique (SST) is an alternative to the conventional double-stapling technique (DST), particularly in low rectal surgery. This systematic review and meta-analysis compared anastomotic leak (AL) between SST and DST.

**Methods:**

A systematic review of the PubMed/MEDLINE, Google Scholar^®^, and Scopus databases was conducted in accordance with PRISMA guidelines from inception through December 2025. The primary outcome of interest was AL. A random-effects meta-analysis was used to compare AL, blood loss, operative time, and length of hospital stay between SST and DST. Risk ratios (RRs) were calculated for dichotomous outcomes and mean differences or standardized mean differences were calculated for continuous outcomes. Sensitivity and subgroup analyses were conducted according to surgical approach. Risk of bias was assessed, and the certainty of the evidence for AL was evaluated using the GRADE framework.

**Results:**

Of 448 articles screened, 14 were included (2 randomized, 12 observational studies), comprising 1326 patients in the SST group and 1720 in the DST group. SST was associated with a significantly lower risk of AL than DST (RR 0.61; 95% confidence interval 0.42 to 0.90; *P* = 0.012), with moderate heterogeneity. Sensitivity analysis excluding studies with zero events yielded consistent results. Subgroup analyses showed no difference in AL for open intracorporeal SST compared with DST, a trend toward benefit with minimally invasive intracorporeal SST compared with DST, and a pronounced reduction in AL with transanal SST compared with DST.

**Conclusions:**

Although the causes of AL are multifactorial, with respect to the stapling technique, SST was associated with a lower risk of AL than DST, and this effect was primarily driven by transanal SST. However, the certainty of evidence is low, and ongoing prospective studies will better define the role of SST.

## Teaser

In a systematic review and random-effects meta-analysis of 14 studies (3046 patients), the single-stapling technique was associated with a lower risk of anastomotic leak than the double-stapling technique (risk ratio 0.61; 95% confidence interval 0.42 to 0.90). This effect appeared to be primarily driven by a transanal single-stapling technique. The certainty of evidence was low, warranting confirmation in prospective studies.

## Introduction

The advent of surgical stapling devices represented a major milestone in colorectal surgery, enabling safe and reproducible low pelvic anastomoses. In the late 1980s and early 1990s, the single-stapling technique (SST), based on an intracorporeal distal purse-string suture and circular stapled anastomosis, was widely adopted with acceptable outcomes^1,2^. However, the technical difficulty of placing a secure purse-string suture in the distal rectum, particularly in low tumours, in a narrow male pelvis, or in patients with obesity, limited its broader applicability and prompted further innovation. The double-stapling technique (DST), introduced by Knight and Griffen in 1980, rapidly became the dominant approach, simplifying pelvic reconstruction by combining linear rectal transection with circular stapled anastomosis^3^. DST reduced operative complexity, minimized contamination, facilitated minimally invasive surgery, and allowed more distal rectal transection, ultimately becoming the standard technique worldwide.

Despite these advances, the reported rate of anastomotic leak (AL) after rectal cancer surgery remains substantial, typically ranging from 5% to 15% and exceeding 20% in high-risk populations^4–6^. This apparent lack of improvement must be interpreted in the context of an evolving and increasingly complex surgical population. The widespread adoption of neoadjuvant chemoradiation therapy (NCRT), expansion of surgical indications to older and more co-morbid patients, and the feasibility of ultra-low rectal anastomoses enabled by minimally invasive and transanal technologies have collectively increased baseline AL risk in contemporary practice. Nevertheless, variability in AL rates has also been attributed to technical characteristics intrinsic to DST, including multiple linear stapler firings, intersecting staple lines, and lateral ‘dog ears’, which may compromise perfusion and the mechanical integrity of the anastomosis^7^.

Importantly, the persistence of AL despite technological progress suggests that refinements in the anastomotic technique itself may represent a key modifiable factor^8,9^. In the minimally invasive era, technical limitations of pelvic access renewed interest in transanal approaches. Transanal total mesorectal excision (TaTME) was developed to facilitate distal rectal dissection and low anastomosis in challenging anatomy. However, concerns regarding its steep learning curve, procedure-specific complications, and oncological safety, particularly following reports of unexpected local recurrence patterns, have limited its widespread adoption and led to a temporary pausing of the technique in several regions^10–12^.

More recently, transanal transection single-stapled anastomosis (TTSS) has emerged as a technical refinement that combines transanal distal rectal transection with a single circular stapled anastomosis while maintaining conventional abdominal total mesorectal excision^13^. By eliminating intersecting staple lines and distal dog ears, TTSS aims to address technical limitations inherent to DST without the complexity of full TaTME. Early studies conducted within the IDEAL framework suggest a lower AL rate with TTSS than DST, although the available evidence remains limited and is predominantly from non-randomized studies^14^. Despite growing interest in SST-based approaches, direct evidence comparing SST and DST remains fragmented. Recent consensus initiatives, including the CoReAL project, have highlighted technical factors such as multiple stapler firings as potential contributors to AL, yet the independent impact of anastomotic configuration has not been systematically evaluated^15^.

The aim of this systematic review was to compare the AL rate between SST and DST in patients undergoing resection for rectal cancer. Secondary outcomes were operative time, intraoperative blood loss, and length of hospital stay.

## Methods

### Study design

This systematic review aimed to evaluate whether SST is associated with a lower rate of AL compared with DST in patients undergoing surgery for rectal cancer. The review protocol was developed according to the PICOS framework (*Table S1*). Eligible studies included those comparing SST with DST in patients undergoing anterior or low anterior resection for rectal or rectosigmoid pathology with respect to AL. Studies focusing exclusively on end colostomy or coloanal anastomosis were excluded. When available, studies including rectal cancer were prioritized; however, studies with mixed malignant and benign indications (for example, diverticular disease) were also included when outcomes for the relevant anastomotic techniques were not reported separately. SST was considered the intervention and DST the comparator. The study protocol was registered with PROSPERO (ID: CRD420251249636).

### Search strategy

This systematic review was conducted in accordance with the PRISMA guidelines, with searches conducted in the PubMed/MEDLINE, Google Scholar^®^, and Scopus databases from inception to December 2025^16^. A comprehensive literature search was performed independently by two reviewers D.K. and C.L. Medical Subject Headings (MeSH) terms and free-text keywords were combined using Boolean operators. The search strategy was as follows: (‘rectal cancer’ OR ‘rectal’) AND (‘anastomotic leak’ OR ‘leak’ OR ‘leakage’) AND (‘single stapled’ OR ‘single-stapled’ OR ‘single-stapling’) AND (‘double stapled’ OR ‘double-stapled’ OR ‘double-stapling’). The full search strategy is detailed in the study protocol. After the removal of duplicates, article titles and abstracts were screened for eligibility. Full-text articles of potentially relevant studies were subsequently retrieved and assessed against the predefined inclusion and exclusion criteria. Reference lists of included studies were manually screened using a snowballing approach to identify additional relevant articles. When full texts could not be retrieved, the authors were contacted. Study selection was performed independently by two authors (D.K. and C.L.), with disagreements resolved through discussion and, when necessary, consultation with a third author (J.T.).

### Data extraction

Data were independently extracted from the full texts of all included studies using predefined, standardized data extraction forms. Extracted study characteristics included first author, country of origin, year of publication, and study design. Details of the surgical approach (open, laparoscopic, or robotic) were recorded, along with the total number of patients in each study arm and whether AL was specified as a primary endpoint. Technical details, including the number of firings in DST and methods for the distal purse-string suture in SST, were also extracted.

Patient-related variables extracted included age, sex, American Society of Anesthesiologists grade, and the use of NCRT. Tumour-related characteristics were also extracted, including pathological stage, reported according to either the tumour node metastasis (TNM) staging system or Dukes classification. In addition, whether the study population consisted exclusively of malignant cases or included benign cases was recorded. Information on the distance of the anastomosis from the anal verge was extracted where available, because this represents a clinically relevant confounding factor for the occurrence of AL.

Outcome data were extracted separately for each predefined endpoint. For AL, the number of patients experiencing AL in each group was recorded. Owing to heterogeneity in outcome definitions, both clinically evident and radiologically detected AL were considered. Data on operative time, intraoperative blood loss, and length of hospital stay were extracted as reported. Where available, *P* values for between-group comparisons of outcomes and the distance of the anastomosis from the anal verge were also extracted. Funding sources and conflicts of interest reported in the included studies were reviewed when available.

When relevant data were missing, incomplete, or reported in a non-extractable format, the information was recorded as not available and no attempt was made to contact study authors. Discrepancies between authors (D.K. and C.L.) during the data extraction process were resolved by discussion and consensus, with involvement of a third author (J.T.) when necessary.

### Outcomes of interest

The primary outcome of interest was the incidence of AL, defined as any clinical or radiological evidence of a leak. Secondary outcomes included operative time, intraoperative blood loss, and the length of hospital stay. Both randomized and non-randomized comparative studies were eligible for inclusion. Single-arm studies, case series, case reports, conference abstracts, reviews, and non-English-language publications were excluded.

### Quality assessment

To minimize the risk of bias, the methodological quality of the included studies was independently assessed. Randomized clinical trials (RCTs) were evaluated using the Cochrane Risk of Bias 2 (RoB-2) tool^17^. Non-randomized comparative studies were assessed using the revised Risk Of Bias In Non-randomized Studies—of Interventions (ROBINS-I) tool (version 2), which evaluates bias across six domains^18^. Each domain was judged as having a low, moderate, serious, or critical risk of bias. An overall risk-of-bias judgment was subsequently derived for each study by integrating the domain-level assessments, and an estimate of the systematic review's overall risk of bias was calculated.

### Certainty of evidence assessment

The certainty of evidence for AL was evaluated using the GRADE framework^19^. The assessment considered the predefined GRADE domains, including risk of bias, indirectness, inconsistency, imprecision, and publication bias, in conjunction with study design. An overall certainty-of-evidence rating was subsequently assigned only for the primary outcome, AL. The GRADE assessment was performed independently by two authors (D.K. and C.L.), with disagreements resolved through discussion and, when necessary, consultation with a third author (J.T.).

### Statistical analysis

Study characteristics were summarized in tabular form. Patient demographics and tumour-related variables are presented separately. Where appropriate, a formal meta-analysis was performed to quantitatively synthesize outcomes between the SST and DST groups. A random-effects meta-analysis was applied for all pooled analyses to account for anticipated clinical and methodological heterogeneity. For dichotomous outcomes, pooled risk ratios (RRs) with their corresponding 95% confidence interval (c.i.) were calculated. For continuous outcomes, pooled effect estimates were calculated using either mean differences (MDs) or standardized mean differences (SMDs), as appropriate. Statistical significance was set at two-sided *P* < 0.05. Statistical heterogeneity was assessed using the *I*^2^ statistic, with values of 25%, 50%, and 75% representing low, moderate, and high heterogeneity, respectively.

When studies reported means and ranges without the standard deviations (s.d.), the s.d. was estimated using the method described by Wan *et al.*^20^. For studies reporting medians and interquartile ranges, mean values were estimated using the method proposed by Luo *et al*., and SDs were estimated using the method proposed by Wan *et al*., in line with current Cochrane recommendations^20,21^. Sensitivity analyses were performed to assess the robustness of the findings. These included the exclusion of studies with zero events in the AL meta-analysis and the exclusion of studies with extreme skewness or outlying values, particularly for length of hospital stay. Prespecified subgroup analyses for AL were conducted according to surgical approach (open, laparoscopic or robotic, and transanal) to explore potential sources of heterogeneity. Publication bias was assessed visually using funnel plots and statistically using Egger’s and Begg’s tests, with *P* < 0.05 considered indicative of small study effects.

## Results

### Study characteristics

**Fig. 1:**
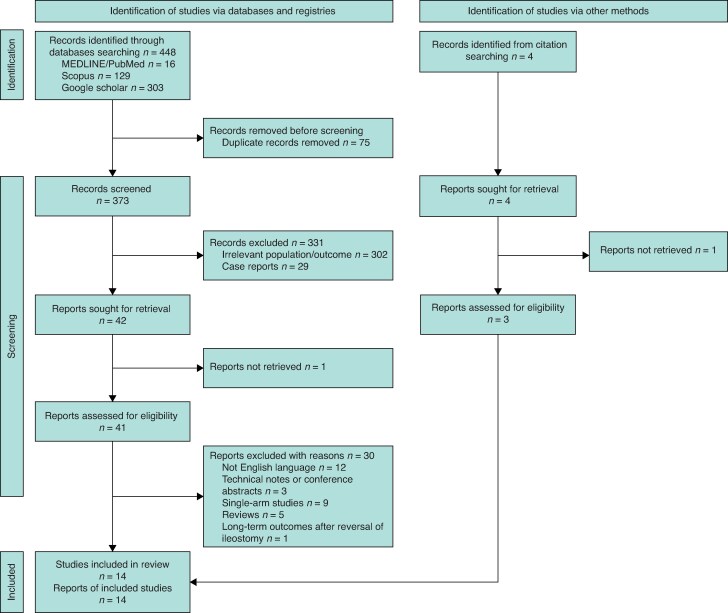
PRISMA flow chart of studies

Following application of the predefined search strategy, 448 records were identified. After the removal of duplicates, title and abstract screening yielded 41 studies eligible for full-text assessment. Application of the predefined inclusion and exclusion criteria resulted in the inclusion of 11 studies. An additional three relevant studies were identified through snowballing, bringing the total to 14 studies included in the final qualitative and quantitative synthesis, as illustrated in the PRISMA flowchart^2,14,22–33^ (*Fig. 1*).

Among the 14 included studies, two were RCTs, five were retrospective cohort studies, and seven were prospective observational studies. The studies were published between 1991 and 2025. Overall, the SST group comprised 1326 patients, whereas the DST group included 1720 patients. Six studies reported only open surgical procedures, whereas eight included minimally invasive approaches. Of the latter, four included only laparoscopic procedures, three included both laparoscopic and robotic procedures, and one study consisted exclusively of robotic procedures.

Reporting of technical aspects of the DST was inconsistent. The number of stapler firings was explicitly reported in five studies^2,14,22,27,29^: three studies reported a single firing, whereas two studies reported mean(s.d.) firings of 1.38(0.53) and 2.42(0.65). With regard to SST, heterogeneity was observed in the technique used for distal purse-string suture placement. In ten studies, the distal purse-string suture was placed intracorporeally in the rectal stump, using an open, laparoscopic, or robotic approach^2,22–25,27–29,32,33^. In one study, the distal purse-string suture was placed following transanal pull-through^26^. In three studies, transanal rectal transection with a distal purse-string suture using either TTSS or TaTME was performed^14,30,31^. AL was set as the primary endpoint in seven studies^14,22,27,29–31,33^ (*Table 1*).

**Table 1:** Characteristics of included studies comparing DST to SST

Author	Country	Year	Study design	Approach	DST		SST		Primary endpoint
					*n*	No. of firings	*n*	Distal purse string suture technique	
Moritz *et al*.^22^	Austria	1991	RCT	Open	35	1	35	Intracorporeal	AL
Bozzetti *et al*.^2^	Italy	1992	Retrospective	Open	49	1	94	Intracorporeal	
Moore *et al*.^23^	Australia	1996	Prospective	Open	65		235	Intracorporeal	
Shrikhande *et al*.^24^	India	2007	Retrospective	Open	138		78	Intracorporeal	
Kim *et al*.^25^	South Korea	2013	Prospective	Laparoscopic Robotic	120		60	Intracorporeal	
Bie *et al.*^26^	China	2013	Prospective	Open	86		45	Transanal pull-through	
Radovanovic *et al.*^27^	Serbia	2014	RCT	Open	50	1	50	Intracorporeal	AL
Saurabh *et al.*^28^	Taiwan	2017	Retrospective	Laparoscopic	106		82	Intracorporeal	
Spinelli *et al.*^14^	Italy	2021	Prospective	Laparoscopic	127	1.38(0.53)*	150	TTSS (50) TaTME (100)	AL
Brunner *et al.*^29^	Germany	2022	Retrospective	Laparoscopic Robotic	141		131	Intracorporeal	AL
Foppa *et al.*^30^	Italy	2023	Prospective	Laparoscopic	458		185	TTSS TaTME	AL
Harji *et al.*^31^	France	2023	Prospective	Laparoscopic Robotic	110		70	TTSS	AL in 30 days
Raju *et al*.^32^	Australia	2025	Retrospective	Laparoscopic	139		40	Intracorporeal	
Filho *et al.*^33^	Brazil	2025	Prospective	Robotic	96	2.42(0.65)*	71	Intracorporeal	AL in 90 days

^*^Mean(standard deviation). DST, double-stapling technique; SST, single-stapling technique; RCT, randomized clinical trial; AL, anastomotic leak; TTSS, transanal transection and single-stapled anastomosis; TaTME, transanal total mesorectal excision; *n*, number of patients.

### Patient characteristics

Baseline patient and tumour characteristics of the included studies are summarized in *Table S2*. Across studies, patients in the SST and DST groups were broadly comparable with respect to age, sex, and American Society of Anesthesiologists grade. The use of NCRT was reported in seven studies and was balanced between the two arms^14,25,27,29–31,33^. Three studies also included benign indications, most commonly diverticular disease; however, malignant pathology accounted for most indications^22,29,32^. Tumour staging, reported using either the TNM or Dukes classification system, was comparable between the SST and DST groups across the included studies.

Reporting of the distance of the anastomosis from the anal verge was heterogeneous. Eight of the 14 studies provided quantitative data on anastomotic level, reported as the mean with s.d. or derived from reported ranges^14,24,25,27,29–31,33^ (*Table 2*). Among these studies, several reported no statistically significant difference in anastomotic height between SST and DST. However, four studies demonstrated a significant difference between groups, with SST performed at a lower or, less commonly, a higher mean distance from the anal verge than DST^25,29,31,33^. Studies reporting a higher anastomotic level in the SST group predominantly used intracorporeal placement of the distal purse-string suture, whereas studies using a transanal purse-string suture approach more frequently reported SST anastomoses at a lower level. Overall, these findings highlight heterogeneity in operative technique and patient selection among the included studies.

**Table 2:** Distance of anastomosis from the anal verge with the DST and SST

Author	Year	Distance from anal verge (cm)				P*
		DST		SST		
		*n*	Mean(s.d.)	*n*	Mean(s.d.)	
Moritz *et al*.^22^	1991	35		35		
Bozzetti *et al*.^2^	1992	49		94		
Moore *et al*.^23^	1996	65		235		
Shrikhande *et al*.^24^	2007	138	7.6(1.58)	78	8.0(1.83)	> 0.05
Kim *et al*.^25^	2013	120	4.4(0.83)	60	4(0.66)	< 0.05
Bie *et al.*^26^	2013	86		45		
Radovanovic *et al.*^27^	2014	50	8(1.16)	50	9(1.33)	0.127
Saurabh *et al.*^28^	2017	106		82		
Spinelli *et al.*^14^	2021	127	6.2(1.86)	150	5.6(2.42)	> 0.05
Brunner *et al.*^29^	2022	141	8(2.16)	131	9(2.16)	0.004
Foppa *et al.*^30^	2023	458	5(1.48)	185	4.66(2.22)	> 0.05
Harji *et al.*^31^	2023	110	5(0.5)	70	3.75(0.5)	< 0.05
Raju *et al.*^32^	2025	139		40		
Filho *et al.*^33^	2025	96	6.27(2.11)	71	5.08(2.32)	< 0.05

DST, double-stapling technique; SST, single-stapling technique; *n*, number of patients. *P value as extracted from each study.

### Anastomotic leak

**Fig. 2:**
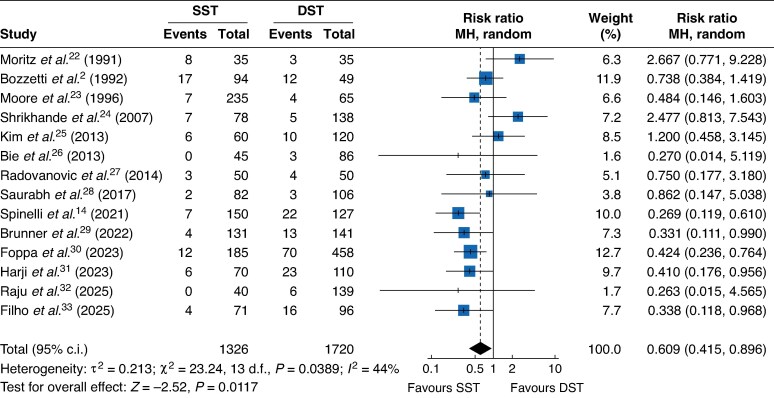
Primary analysis: forest plot comparing anastomotic leak between SST and DST Values in parentheses are 95% confidence intervals. SST, single-stapling technique; DST, double-stapling technique; M-H, Mantel–Haenszel.

**Fig. 3:**
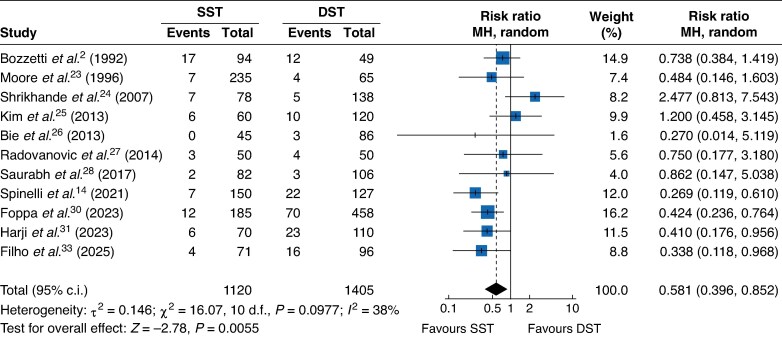
Sensitivity analysis: forest plot comparing anastomotic leak, excluding mixed cohorts (benign/malignant), between SST and DST Values in parentheses are 95% confidence intervals. SST, single-stapling technique; DST, double-stapling technique; M-H, Mantel–Haenszel.

Fourteen studies encompassing 3046 patients were included in the primary meta-analysis of AL, with 1326 patients in the SST group and 1720 patients in the DST group^2,14,22–33^. Pooled analysis using random-effects meta-analysis demonstrated a significantly lower risk of AL in the SST group compared with the DST group (RR 0.61; 95% c.i. 0.42 to 0.90; *P* = 0.012). Moderate heterogeneity was observed among studies (*I*^2^ = 44%). Two studies reported zero events in the SST arm^26,32^ (*Fig. 2*). Sensitivity analysis excluding these two studies yielded consistent results, with no material change in the direction or magnitude of the pooled effect estimate (RR 0.63; 95% c.i. 0.419 to 0.945; *P* = 0.025). Between-study heterogeneity remained comparable (*I*^2^ = 51%). The corresponding forest plot is shown in *Fig. S1*. To account for potential heterogeneity related to study indication, a sensitivity analysis excluding studies that included benign or mixed pathology was performed. The pooled analysis including malignancy-only cohorts (11 studies) demonstrated that the primary signal favouring SST for reduction of AL remained statistically significant, with moderate heterogeneity (*I*^2^ = 38%; *Fig. 3*).

**Fig. 4:**
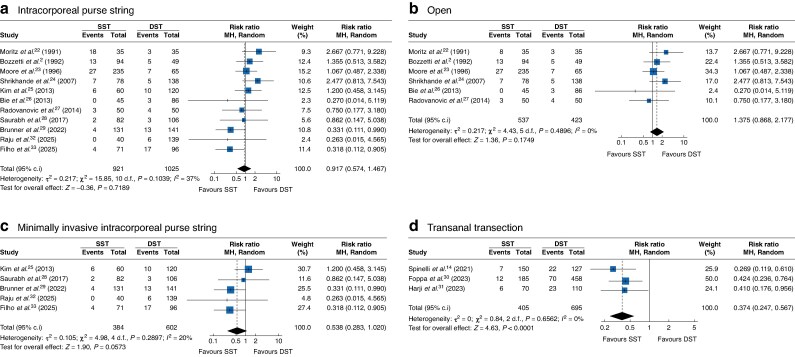
Subgroup analyses: forest plots comparing AL between SST and DST stratified according to surgical approach **a** Intracorporeal purse string; **b** open; **c** minimally invasive intracorporeal purse string; and **d** transanal transection. Values in parentheses are 95% confidence intervals. AL, anastomotic leak; SST, single-stapling technique; DST, double-stapling technique; M-H, Mantel–Haenszel.

Subgroup analyses stratified by surgical approach were performed to evaluate the consistency of the primary outcome across different technical settings. In studies using an intracorporeal purse-string technique, no significant difference in AL was observed between SST and DST (11 studies; RR 0.92; 95% c.i. 0.57 to 1.47; *I*^2^ = 37%; *Fig. 4a*). Similarly, no statistically significant difference was identified in the intracorporeal minimally invasive subgroup, although the effect estimate favoured SST and approached significance (5 studies; RR 0.54; 95% c.i. 0.28 to 1.02; *P* = 0.0573; *I*^2^ = 20%; *Fig. 4c*). In contrast, open intracorporeal SST was not associated with a reduction in AL compared with DST (6 studies; RR 1.38; 95% c.i. 0.87 to 2.18; *I*^2^ = 0%; *Fig. 4b*). A significantly lower risk of AL was observed in the transanal SST subgroup (3 studies; RR 0.37; 95% c.i. 0.25 to 0.57; *I*^2^ = 0%; *P* < 0.0001), representing the most pronounced effect among all subgroups (*Fig. 4d*).

Publication bias for AL was assessed. Visual inspection did not demonstrate relevant funnel plot asymmetry (*Fig. S2*). Neither Egger’s test (*P* = 0.619) nor Begg’s test (*P* = 0.250) indicated evidence of small study effects.

### Perioperative outcomes

Estimated intraoperative blood loss was reported in two studies only, both using intracorporeal purse-string suture placement and minimally invasive (laparoscopic or robotic) approaches^28,29^. Pooled analysis using SMD demonstrated a small but statistically significant reduction in blood loss associated with SST compared with DST (SMD −0.23; 95% c.i. −0.42 to −0.05; *P* = 0.013), with no observed heterogeneity (*I*^2^ = 0%). The prediction interval was wide, and given the limited number of contributing studies and the use of a standardized effect measure, the clinical relevance of this finding remains uncertain (*Fig. S3*).

Operative time was reported in eight studies, comprising 654 patients in the SST group and 1222 patients in the DST group^14,22,25,28–30,32,33^. Pooled analysis demonstrated a trend towards shorter operative time with DST than SST; however, this difference did not reach statistical significance (MD 11.3 minutes; 95% c.i. −0.4 to 22.9  minutes; *P* = 0.058). Substantial between-study heterogeneity was observed (*I*^2^ = 72%). Given the wide c.i. and high heterogeneity, these findings should be interpreted with caution (*Fig. S4*).

The length of hospital stay was reported in eight studies^14,24,25,28–33^. However, two studies were excluded from the quantitative synthesis due to extreme skewness in reported values^30,33^. Pooled analysis of the remaining six studies, comprising 533 patients in the SST group and 743 patients in the DST group, demonstrated a significantly shorter length of hospital stay associated with SST (MD −0.77 days; 95% c.i. −1.35 to −0.18 days; *P* = 0.010). No between-study heterogeneity was observed (*I*^2^ = 0%; *Fig. S5*).

### Quality assessment of studies

**Fig. 5:**
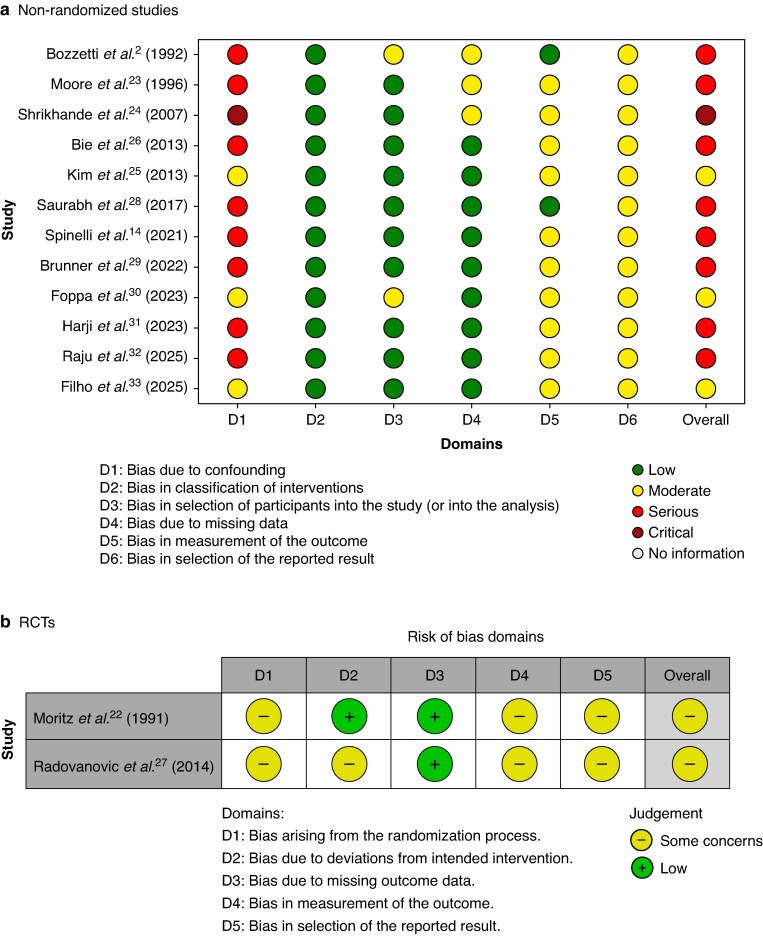
Risk of bias assessment of the included studies **a** Risk of bias assessment of non-randomized studies using the ROBINS-I v2 tool. **b** Risk of bias assessment of RCTs using the RoB-2 tool. ROBINS-I v2, Risk Of Bias In Non-randomized Studies – of Interventions; RCTs, randomized clinical trials; RoB-2 tool, revised tool for Risk of Bias in randomized trials.

Risk of bias in non-randomized studies was assessed using the ROBINS-I tool, which indicated an overall serious risk of bias across the 12 observational studies (*Fig. 5a*). The two RCTs were assessed using the RoB-2 tool and were judged to have some concerns (*Fig. 5b*).

### Certainty of evidence assessment

**Fig. 6:**
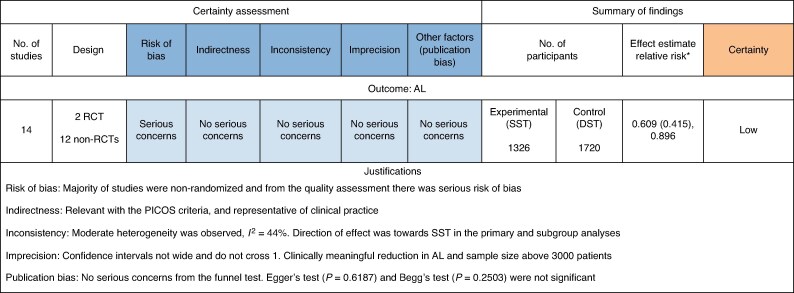
GRADE assessment of studies of AL AL, anastomotic leak; RCT, randomized clinical trial; SST, single-stapling technique; DST, double-stapling technique.

The certainty of evidence was assessed using the GRADE approach for AL. Given that the body of evidence consisted predominantly of observational studies and was judged to have a serious risk of bias, the certainty of evidence was downgraded to low. No serious concerns were identified for indirectness, imprecision, inconsistency, or publication bias. Consequently, the overall certainty of evidence for the effect of SST *versus* DST on AL was rated as low (*Fig. 6*).

## Discussion

Anastomotic technique represents a potentially modifiable factor that may influence the rate of AL in rectal cancer surgery. Although SST is not a novel concept, it has regained interest following the widespread adoption of DST, particularly in the context of transanal rectal resection, where the technical constraints of DST may be more pronounced. This systematic review and meta-analysis primarily compared the AL rate between SST and DST and, secondarily, evaluated perioperative outcomes, including operative time, length of hospital stay, and intraoperative blood loss. Pooled analysis demonstrated an overall reduction in AL associated with SST compared with DST; however, subgroup analyses indicated that this effect was not uniform across SST implementations and was largely driven by the transanal SST subgroup. However, the overall certainty of the evidence was rated as low, primarily due to a serious risk of bias associated with the observational design of most included studies. Regarding perioperative outcomes, SST was associated with a modest reduction in the length of hospital stay and a small standardized reduction in intraoperative blood loss, whereas operative time tended to be shorter with DST, although this difference did not reach statistical significance. These secondary findings were based on a limited number of studies and should be interpreted cautiously.

Although the overall pooled analysis demonstrated a significantly lower AL rate with SST, subgroup analyses stratified by surgical approach provided important insights into the context in which this benefit is most apparent. In studies using an intracorporeal purse-string suture technique, no significant difference in AL was observed between SST and DST. This finding is clinically plausible, because intracorporeal purse-string suture placement generally requires a sufficiently long distal rectal stump and is therefore more feasible in middle or upper rectal resections, where baseline AL rates are lower and technical constraints are less pronounced^29^. Consistent with this, several studies using intracorporeal purse-string SST reported anastomoses performed at relatively higher distances from the anal verge, either explicitly or by applying a predefined cut-off (for example, ≥ 10 cm), suggesting the inclusion of predominantly anterior resections^2,22,23,32^. In these settings, differences between stapling techniques are less likely to translate into measurable differences in AL. In a further subgroup analysis restricted to intracorporeal purse-string SST *versus* DST, minimally invasive approaches (robotic or laparoscopic) demonstrated a trend towards lower AL rates, whereas open intracorporeal SST was not associated with a reduction. These findings likely reflect improved visualization and instrument control with minimally invasive techniques compared with the technical challenges of purse-string suture placement in a short rectal stump using an open approach^33^. Nevertheless, the benefit of SST was mostly pronounced in studies using a transanal approach, where a significantly lower AL rate was observed (*P* < 0.0001). Transanal rectal transection facilitates direct visualization and controlled purse-string placement at very low anastomotic levels, thereby overcoming the anatomical limitations of the narrow pelvis. Together, these findings suggest that the observed reduction in AL with SST is driven primarily by its application in low rectal anastomoses, where technical challenges are greatest and baseline leak risk is highest, rather than by intrinsic differences between stapling configurations in higher rectal resections.

Differences in perioperative outcomes between SST and DST were modest and should be interpreted cautiously. Operative time showed a non-significant trend favouring DST, accompanied by substantial heterogeneity across studies. Given the small absolute difference observed and the fact that operative duration is affected by multiple factors, this finding is unlikely to be clinically meaningful in most settings. Operative time is strongly influenced by factors such as surgical approach and procedural complexity; for example, TaTME is inherently more time-consuming due to the perineal phase, whereas multiple stapler firings during DST may, conversely, prolong operative duration and increase technical complexity^14,34^. Intraoperative blood loss was reported in only a limited number of studies and demonstrated a small standardized reduction associated with SST. However, because blood loss is primarily determined by preservation of the mesorectal plane rather than anastomotic configuration, and given the restricted technical context and limited number of contributing studies, the clinical relevance of this finding remains uncertain^35^. The length of hospital stay was modestly shorter in the SST group. Although this may partly reflect the lower AL rate observed with SST, the length of hospital stay is influenced by multiple perioperative and institutional factors and therefore represents an indirect outcome^36^. Moreover, quantitative synthesis required the exclusion of studies with extreme skewness, further limiting interpretability. Overall, perioperative outcomes did not demonstrate consistent or clinically meaningful differences between techniques and should be considered supportive rather than determinative findings.

Few studies have reported functional outcomes after SST and DST. Among the included studies, only one evaluated postoperative bowel function using the low anterior resection syndrome (LARS) score. At 12 months, no significant difference was observed between SST and DST in the proportion of patients with a LARS score > 20, indicating minor or major LARS^31^. In another study, Foppa *et al*. reported comparable LARS scores between SST and DST at 6 and 12 months, with a significantly lower median LARS score in the transanal SST cohort at 24 months^37^. Although LARS is multifactorial, with NCRT, and anastomotic height being the most prominent factors, AL is proposed to be one of the most important clinical factors predicting LARS^38^. In a recent systematic review and meta-analysis including 20 retrospective studies comprising 4764 patients, AL was detected in 14% of patients, and those who experienced AL had a higher risk of LARS^39^. In this context, the lower AL rate observed with SST, particularly in low rectal anastomoses, may have important implications for long-term functional outcomes. However, robust conclusions cannot be drawn due to the limited and heterogeneous reporting of functional data, underscoring the need for prospective studies with standardized functional assessment and long-term follow-up.

Several limitations should be considered when interpreting the findings of this systematic review. Distance from the anal verge, a well-established determinant of AL, differed between the SST and DST groups in a subset of studies and likely reflects intrinsic technical differences between the two approaches. SST, particularly via a transanal approach, is more feasible in very low rectal anastomoses, whereas intracorporeal purse-string suture placement was generally performed at higher anastomotic levels. This heterogeneity in anastomotic height may have influenced pooled leak rates and complicated direct comparisons. Importantly, AL as a complication is influenced by many factors, including patient-, disease-, and procedure-related factors beyond stapling technique alone, potentially limiting the interpretability and generalizability of comparisons between SST and DST. Furthermore, important clinical variables that may influence AL, including diversion practices and neoadjuvant therapy, were inconsistently reported across studies, precluding formal meta-regression analyses. The included studies spanned more than three decades, during which substantial changes occurred in stapling technology, the adoption of minimally invasive approaches, perioperative management pathways, diversion practices, and the detection and reporting of AL. These temporal differences likely introduced additional clinical heterogeneity that cannot be fully accounted for in pooled analyses. Most studies were observational and judged to have a serious risk of bias, resulting in low certainty of evidence for the primary outcome. Although sensitivity and subgroup analyses were performed, residual confounding remains likely. Reporting of perioperative outcomes was inconsistent and limited, restricting the robustness of pooled estimates. The required length of hospital stay excluded studies with extreme skewness, whereas operative time and blood loss are indirect outcomes influenced by multiple procedural and institutional factors. Definitions and reporting of AL varied across studies, including differences in diagnostic criteria (clinical *versus* radiological leaks), grading systems, and assessment time windows (for example, 30-day or 90-day outcomes). In several studies, AL was not the primary endpoint, and definitions were not fully specified, raising the potential for outcome misclassification. Moderate heterogeneity was observed for AL, and some studies included benign indications alongside rectal cancer. Finally, overlap among patient cohorts from the same institutions cannot be ruled out entirely.

This study focused on anastomotic and postoperative outcomes and did not evaluate oncological endpoints such as R0 resection rates or lymph node yield. Although these are important considerations, their inclusion would have significantly changed the scope of the analysis and was therefore beyond the focus of this work. It should be noted that surgeon expertise and patient selection likely play a role in outcomes, particularly for technically demanding procedures such as TaTME or TTSS, which have a steep learning curve. These factors may contribute to cautious patient selection and influence reported complication rates.

Despite these limitations, this study has important strengths. To the authors’ knowledge, this is the first systematic review and meta-analysis specifically comparing SST and DST for rectal anastomosis. A comprehensive and transparent methodology was used, including predefined eligibility criteria, systematic risk-of-bias assessment using validated tools, application of the GRADE framework for the primary outcome, and sensitivity and subgroup analyses to explore the robustness of findings and key technical variables.

Several randomized and prospective studies are currently underway to further clarify the comparative outcomes of SST *versus* DST. The OASIS study (NCT07146334), is an international multicentre prospective trial comparing TTSS to DST but has not yet started recruitment. Another study, TTSS-REC (NCT06314646), is being conducted in Italy as an observational IDEAL stage 2b study, aiming to evaluate AL, functional outcomes, including LARS, and oncological recurrence.

This systematic review and meta-analysis suggest that SST may be associated with a lower risk of AL compared with DST in rectal anastomosis. Subgroup analyses indicated that this potential benefit was not uniform across SST implementations: the most pronounced effect was observed in transanal resections, whereas intracorporeal techniques showed no clear advantage. These findings support the concept that any potential benefit of SST may be confined to very low rectal anastomoses, where technical challenges and baseline leak risk are greatest. However, these results should be interpreted cautiously, because residual confounding related to anastomotic height, patient selection, study era, and variability in outcome reporting cannot be excluded. Prospective comparative studies are required to confirm these observations and to better define the role of SST in different clinical contexts.

## Supplementary Material

zrag078_Supplementary_Data

## Data Availability

Data are available from the corresponding author upon reasonable request.
