# Supplementary material for: Single-stapling versus double-stapling technique for rectal anastomosis—meta-analysis
Source: BJS Open. 2026 Jul 3;10(4):zrag078. doi: 10.1093/bjsopen/zrag078 (PMC13331353; doi:10.1093/bjsopen/zrag078)

**Single-stapled versus double-stapled technique for rectal anastomosis – A systematic literature review and meta-analysis**

Dimitrios Kehagias ^1^, Charalampos Lampropoulos ^2^, Ioannis Kehagias ^3^, David Jayne ^1,4^, Jim Tiernan ^1^

^1^ John Goligher Colorectal Unit, Leeds Teaching Hospitals NHS Trust, Leeds, UK

^2^ Intensive Care Unit, St Andrew’s General Hospital of Patras, Patras, Greece

^3^ Department of Surgery, University Hospital of Patras, Patras, Greece

^4^ Leeds Institute of Medical Research, University of Leeds, Leeds, UK

**Corresponding author**

Dimitrios Kehagias MD, PhD, dimikech@gmail.com, ORCID: 0000-0001-7691-6355

**Supplementary Materials - Index**

| **Supplementary Tables** |  |
| --- | --- |
| Table S1. PICOS framework | *page 2* |
| Table S2. Patient characteristics of the included studies | *page 3* |
| **Supplementary Figures** |  |
| Figure S1. Sensitivity analysis - AL, SST vs DST | *page 4* |
| Figure S2. AL - Funnel plot | *page 5* |
| Figure S3. Blood loss - SST vs DST | *page 6* |
| Figure S4. Operative time - SST vs DST | *page 7* |
| Figure S5. Length of stay - SST vs DST | *page 8* |
|  |  |

**Supplementary Tables**

**Table S1.** PICOS framework

|  | **Inclusion criteria** | **Exclusion criteria** |
| --- | --- | --- |
| **Population** | Anterior or low-anterior resection for rectal or rectosigmoid disease (primarily rectal cancer, but mixed malignant/benign cohorts eligible) | End colostomy  Coloanal anastomosis |
| **Intervention** | Open, laparoscopic or robotic SST with intracorporeal purse string, or transanal purse string suture (TTSS or TaTME) | .. |
| **Comparator** | Open, laparoscopic or robotic  DST conventional | .. |
| **Outcome** | Primary: Anastomotic leak  Secondary: Operative time, blood loss,  length of stay | .. |
| **Study design** | Randomized  Non-randomized - prospective or retrospective | Case reports, case series,  single-arm studies |

**SST: single-stapled technique; TTSS: transanal transection single-stapled; TaTME: transanal total mesorectal excision; DST: double-stapled technique**

|  | **Double-stapled technique (DST)** | | | | | | | **Single-stapled technique (SST)** | | | | | | |
| --- | --- | --- | --- | --- | --- | --- | --- | --- | --- | --- | --- | --- | --- | --- |
| **Author** | **N** | **Age** | **Sex** | **ASA** | **NCRT** | **Stage** | **Distance from anal verge (cm)** | **n** | **age** | **Sex** | **ASA** | **NCRT** | **Stage** | **Distance from anal verge (cm)** |
| Moritz et al | 35 | 67.2 | M (16)  F (19) | .. | .. | Comparable Dukes stage  Diverticular cases | ≤ 10 (14)  > 10 (21) | 35 | 65.7 | M (17)  F (18) | .. | .. | Comparable Dukes stage  Diverticular cases | ≤ 10 (15)  > 10 (20) |
| Bozzetti et al | 49 | 60 | M (20)  F (29) | .. | .. | .. | >10 (12)  ≤10 (37) | 94 | 61.5 | M (45)  F (49) | .. | .. | .. | >10 (40)  ≤10 (54) |
| Moore et al | 65 | 66.5 | M (50)  F (15) | .. | .. | comparable | >10 (8)  ≤10 (57) | 235 | 67.6 | M (171)  F (64) | .. | .. | comparable | >10 (72)  ≤10 (163) |
| Shrikhande et al | 138 | 50.3 ± 13.9 | M (90)  F (48) | .. | .. | Comparable Dukes | 7.6 (2.5 – 12)* | 78 | 53.2 ± 13.5 | M (56)  F (22) | .. | .. | Comparable Dukes | 8 (4 – 15) |
| Kim et al | 120 | 62.9 | M (63)  F (57) | I (53)  II (64)  III (3) | 12 | T1 (30)  T2 (24)  T3 (64)  T4 (2) | 7.9 | 60 | 59.7 | M (26)  F (34) | I (29)  II (28)  III (3) | 5 | T1 (16)  T2 (17)  T3 (27)  T4 (0) | 7.8 |
| Bie et al | 86 | 55 | M (40)  F (46) | .. | .. | Comparable Dukes | 4 – 6.5 | 45 | 55 | M (19)  F (26) | .. | .. | Comparable Dukes | 4 – 6.5 |
| Radovanovic et al | 50 | 64 | M (26)  F (24) | I (5)  II (28)  III (17) | 18 | T1 (12)  T2 (10)  T3 (21)  T4 (4) | 8 (4–11) * | 50 | 66 | M (31)  F (19) | I (3)  II (27)  III (20) | 21 | T1 (21)  T2 (7)  T3 (15)  T4 (7) | 9 (4–12) |
| Saurabh et al | 106 | 64.7 | M (65)  F (41) | I, II (75)  III (31) | .. | T1 (6)  T2 (41)  T3 (59) | > 10 (106) | 82 | 63.3 | M (47)  F (35) | I, II (59)  III (23) | .. | T1 (7)  T2 (35)  T3 (40) | > 10 (82) |
| Spinelli et al | 127 | 64.6 | M (68)  F (59) | I (30)  II (77)  III (20) | 76 | Comparable | 6.2 ± 1.86 ^†^ | 150 | 64.3 | M (33)  F (17) | I (14)  II (27)  III (9) | 33 | Comparable | 5.6 ± 2.42 |
| Brunner et al | 141 | 51.6 | M (61)  F (80) | I (30)  II (96)  III (15) | 23 | Benign Malignant | 8 (3−16) * | 131 | 56.9 | M (53)  F (78) | I (14)  II (100)  III (16) | 16 | Benign Malignant | 9 (3−16) |
| Foppa et al | 458 | 64.6 | M (367)  F (191) | I (119)  II (286)  III (50)  IV (3) | 335 | comparable | 5 (4 - 6) * | 185 | 62.7 | M (123)  F (62) | I (65)  II (99)  III (18)  IV (3) | 140 | comparable | 5 (3 - 6) |
| Harji et al | 110 | 65 | M (73)  F (37) |  | 57 | T1–2 (18)  T3–4 (92) | 5 (3–6) * | 70 | 64 | M (43)  F (27) |  | 51 | T1–2 (11)  T3–4 (59) | 3.75 (2–5) |
| Raju et al | 139 | 59.5 | M (57)  F (82) | .. | .. | Benign  Malignant | .. | 40 | 60.9 | M (15)  F (25) | .. | .. | Benign  Malignant | .. |
| Filho et al | 96 | 58.3 | M (61)  F (35) | I (17)  II (70)  III (8)  IV (1) | 65 | I (19)  II (12)  III (61)  IV (4) | 6.27 ± 2.11 ^†^ | 71 | 55.6 | M (51)  F (20) | I (7)  II (50)  III (14)  IV (0) | 38 | I (23)  II (6)  III (41)  IV (1) | 5.08 ± 2.32 |

**Table S2.** Patient characteristics of the included studies.

**^†^ expressed as mean ± standard deviation, * expressed as mean and range**

**Supplementary Figures**

**Figure S1.** Sensitivity analysis - AL, SST vs DST

**
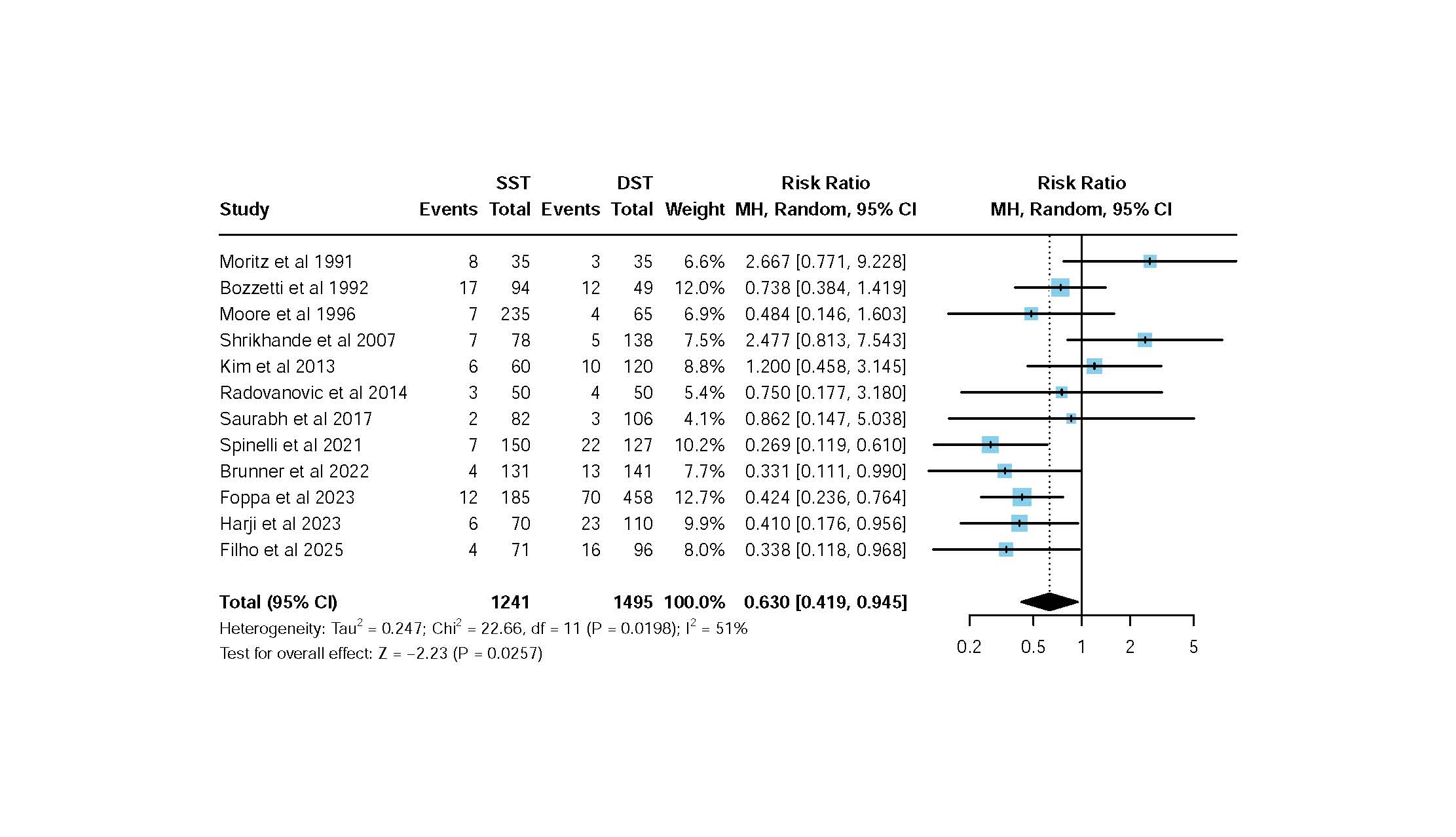
**

**Figure S2.** AL - Funnel plot


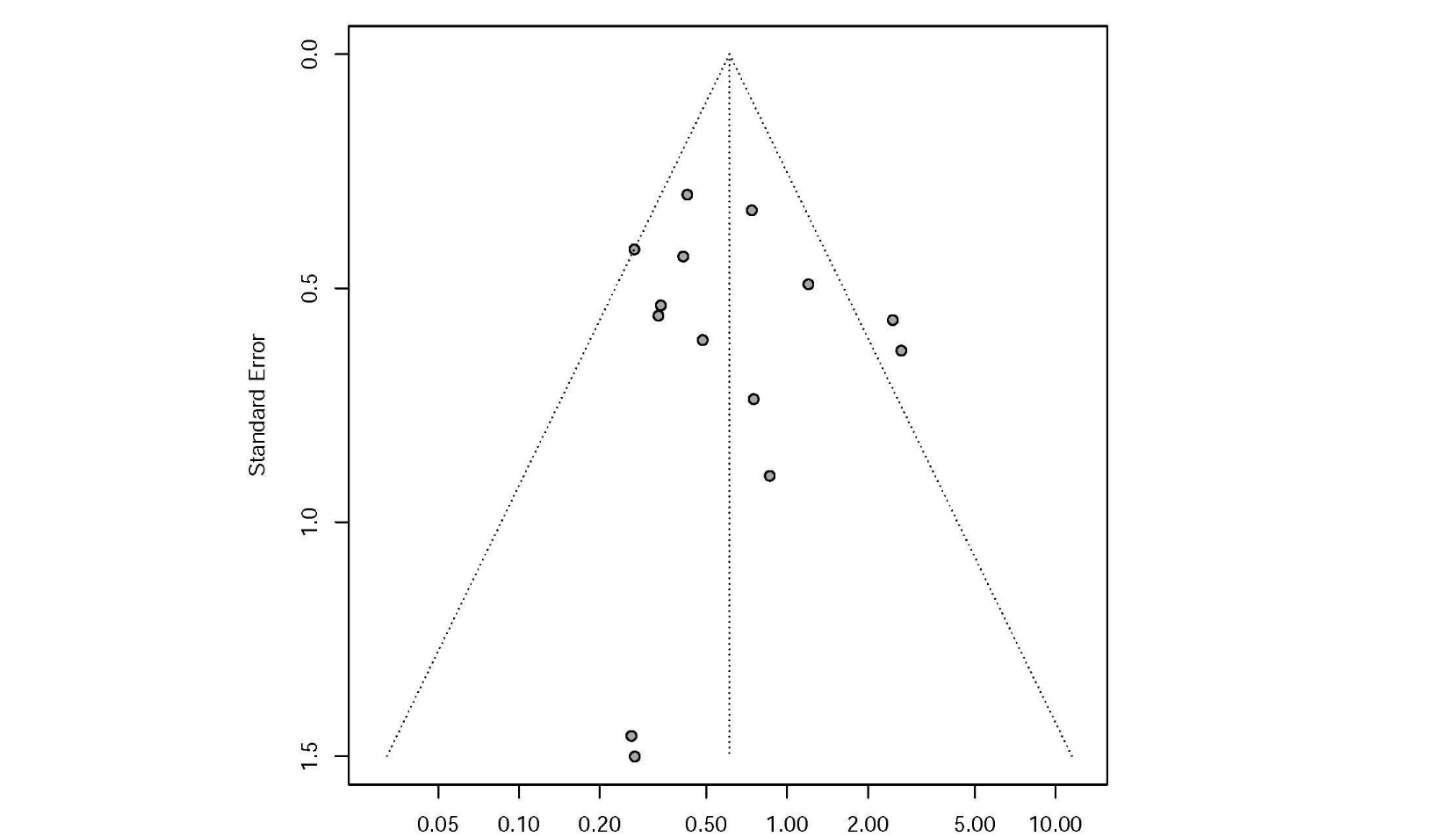


**Figure S3.** Blood loss - SST vs DST


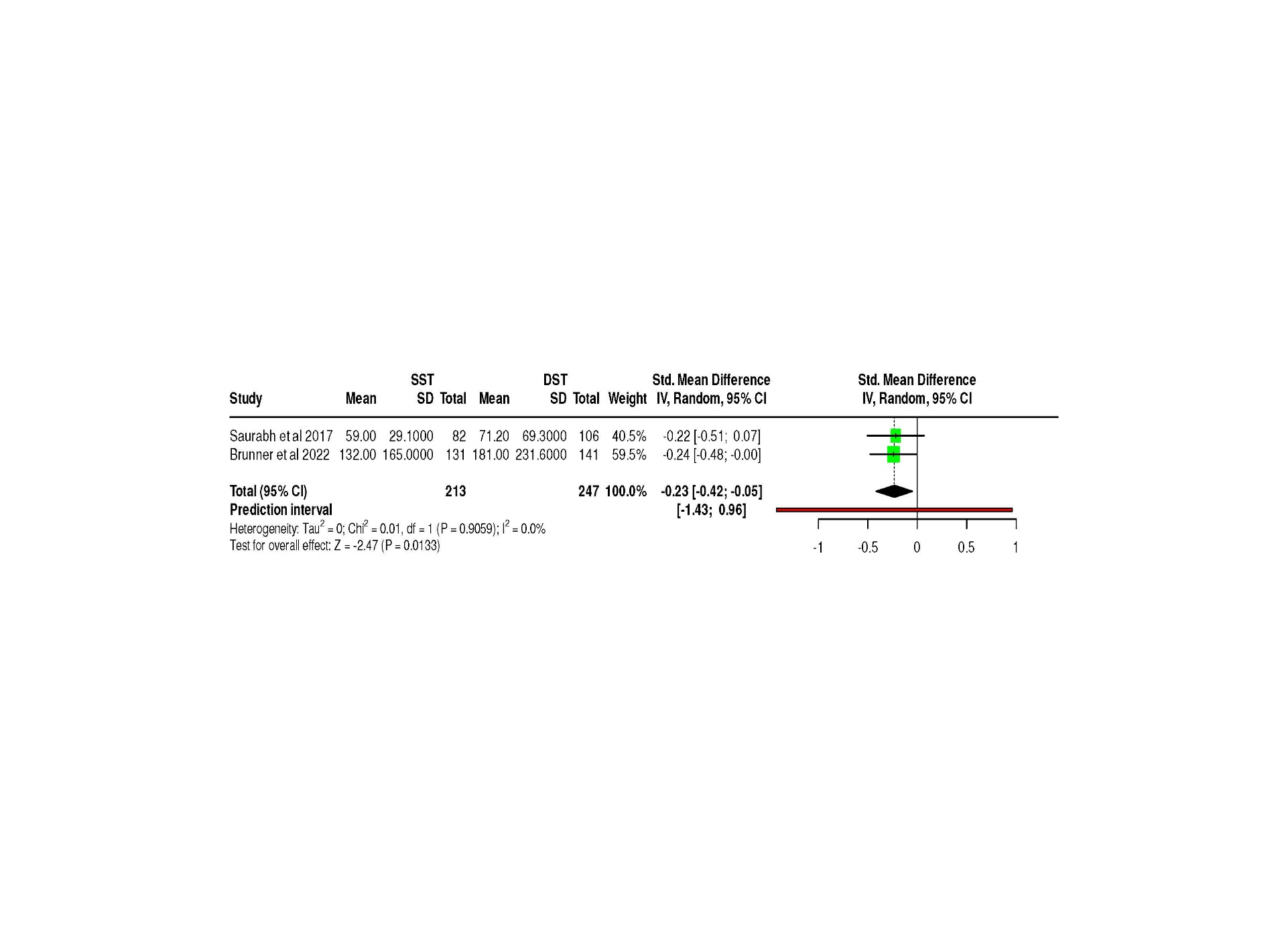


**Figure S4.** Operative time - SST vs DST


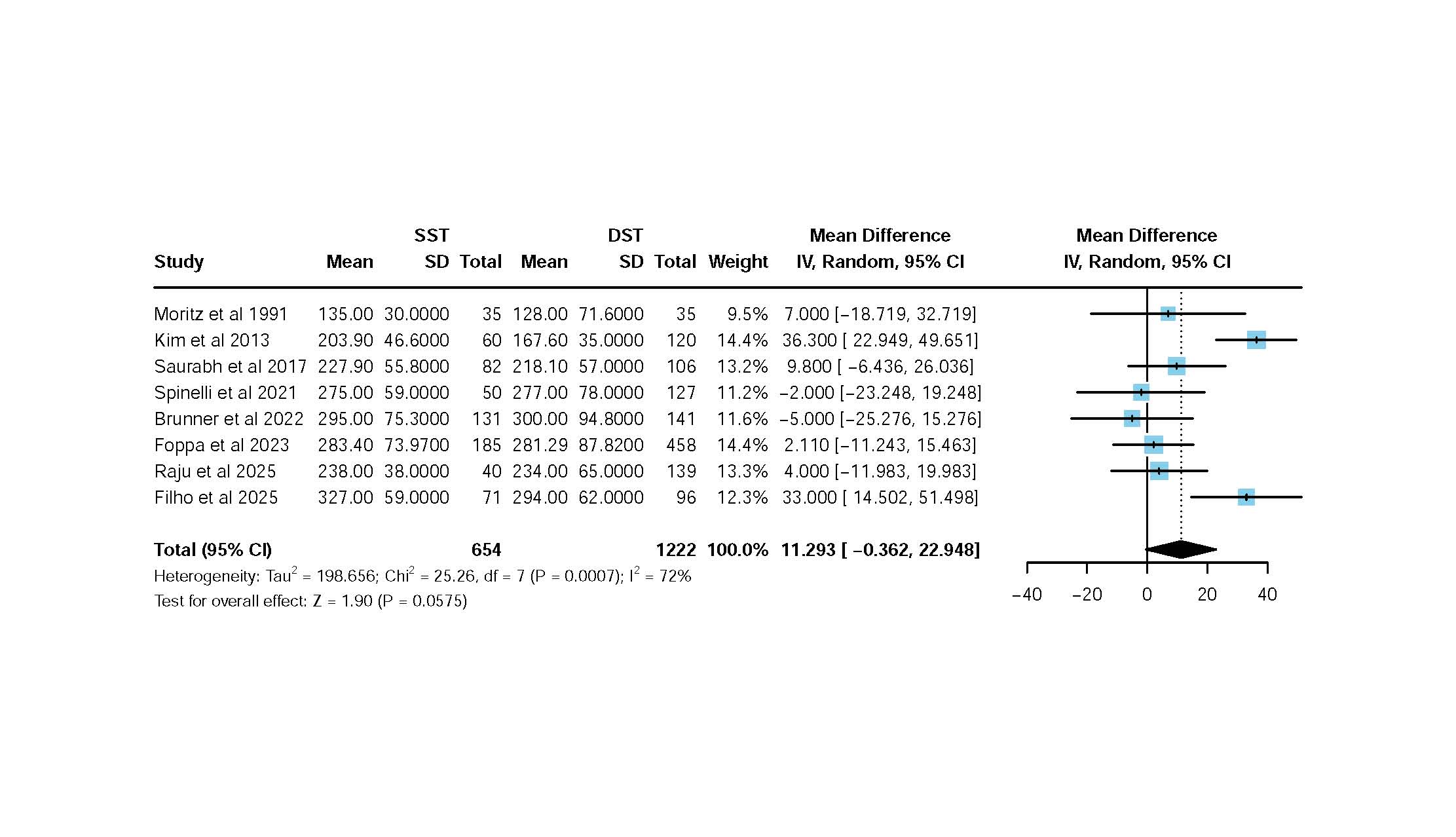


**Figure S5.** Length of stay - SST vs DST


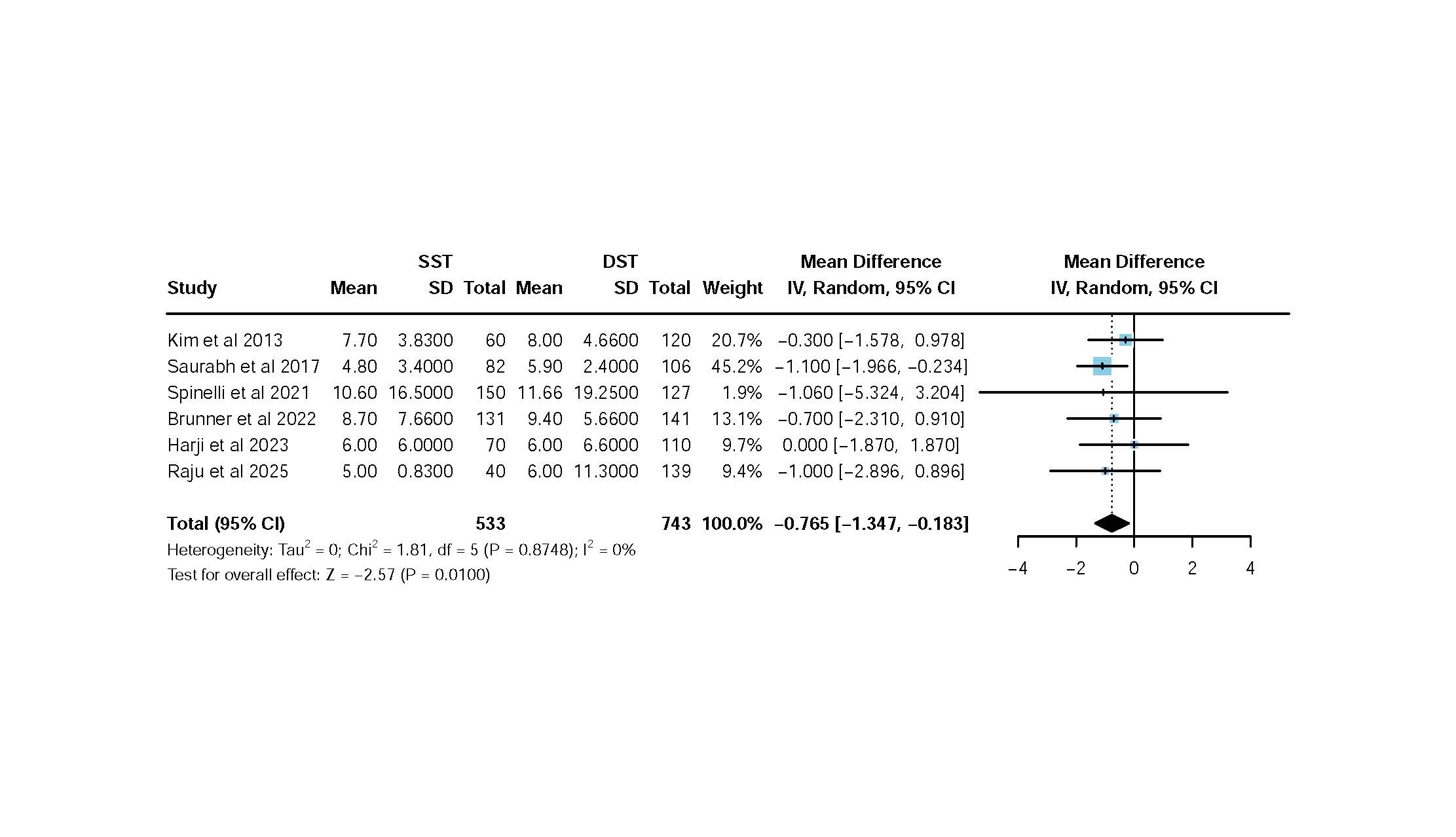

Supplement: zrag078_Supplementary_Data [file zrag078_supplementary_data.zip › Supplementary_Material.docx]
